# Next-generation sequencing for molecular diagnosis of lung adenocarcinoma specimens obtained by fine needle aspiration cytology

**DOI:** 10.1038/srep11317

**Published:** 2015-06-11

**Authors:** Tian Qiu, Huiqin Guo, Huan Zhao, Luhua Wang, Zhihui Zhang

**Affiliations:** 1 Department of Pathology, Cancer Hospital, Peking Union Medical College and Chinese Academy of Medical Sciences, Beijing, China; 2 Department of Radiation Oncology, Cancer Hospital, Peking Union Medical College and Chinese Academy of Medical Sciences, Beijing, China

## Abstract

Identification of multi-gene variations has led to the development of new targeted therapies in lung adenocarcinoma patients, and identification of an appropriate patient population with a reliable screening method is the key to the overall success of tumor targeted therapies. In this study, we used the Ion Torrent next-generation sequencing (NGS) technique to screen for mutations in 89 cases of lung adenocarcinoma metastatic lymph node specimens obtained by fine-needle aspiration cytology (FNAC). Of the 89 specimens, 30 (34%) were found to harbor epidermal growth factor receptor (EGFR) kinase domain mutations. Seven (8%) samples harbored *KRAS* mutations, and three (3%) samples had *BRAF* mutations involving exon 11 (G469A) and exon 15 (V600E). Eight (9%) samples harbored *PIK3CA* mutations. One (1%) sample had a *HRAS* G12C mutation. Thirty-two (36%) samples (36%) harbored *TP53* mutations. Other genes including *APC, ATM, MET, PTPN11, GNAS, HRAS, RB1, SMAD4* and *STK11* were found each in one case. Our study has demonstrated that NGS using the Ion Torrent technology is a useful tool for gene mutation screening in lung adenocarcinoma metastatic lymph node specimens obtained by FNAC, and may promote the development of new targeted therapies in lung adenocarcinoma patients.

Lung cancer is the main cancer in the world today, whether considered in terms of numbers of cases or deaths, because of the high case fatality^1^,^2^,^3^. Non-small cell lung cancer (NSCLC) is the most common type of lung cancer and is often diagnosed at advanced stages of the disease. Lung adenocarcinoma is one of the most common subtypes in NSCLC. Conventional chemotherapeutic regimens have not significantly increased overall survival in the treatment of advanced NSCLC^4^. A growing body of research has focused on the association between specific genomic alterations and response of advanced-stage lung cancer to targeted therapies^5^. Small molecule inhibitors that target oncogenic tyrosine kinases have become an attractive treatment strategy in the therapy of several cancers^6^. Identification of epidermal growth factor receptor (EGFR) kinase domain activating mutations in NSCLC patients led to several retrospective studies to confirm the relationships between the *EGFR* mutational status and treatment response to kinase inhibitors such as erlotinib and gefitinib^7^,^8^,^9^,^10^. Recently, the identification of multiple genetic variations has led to the development of new targeted therapies in a subset of NSCLC patients^11^,^12^. Identification of an appropriate patient population with a reliable screening method is the key to the overall success of tumor targeted therapies.

Most patients with NSCLC are diagnosed at an advanced stage, when many of them have lost the opportunity of surgical treatment and have to be diagnosed by cytological specimens^13^. Currently, Sanger sequencing and real-time PCR have become the clinical methods used to detect gene mutations in diagnostic laboratories. However, they are expensive and time-consuming for a large number of gene detections, thus limiting their clinical applications. The recent commercial availability of the next-generation sequencing (NGS) technique has changed the way we think about scientific approaches in basic, applied and clinical research^14^,^15^,^16^. In this study, we used the Ion Torrent NGS technique to screen for gene mutations in lung adenocarcinoma metastatic lymph node specimens obtained by fine-needle aspiration cytology (FNAC).

## Results

### Performance of next-generation sequencing

**Figure 1:**
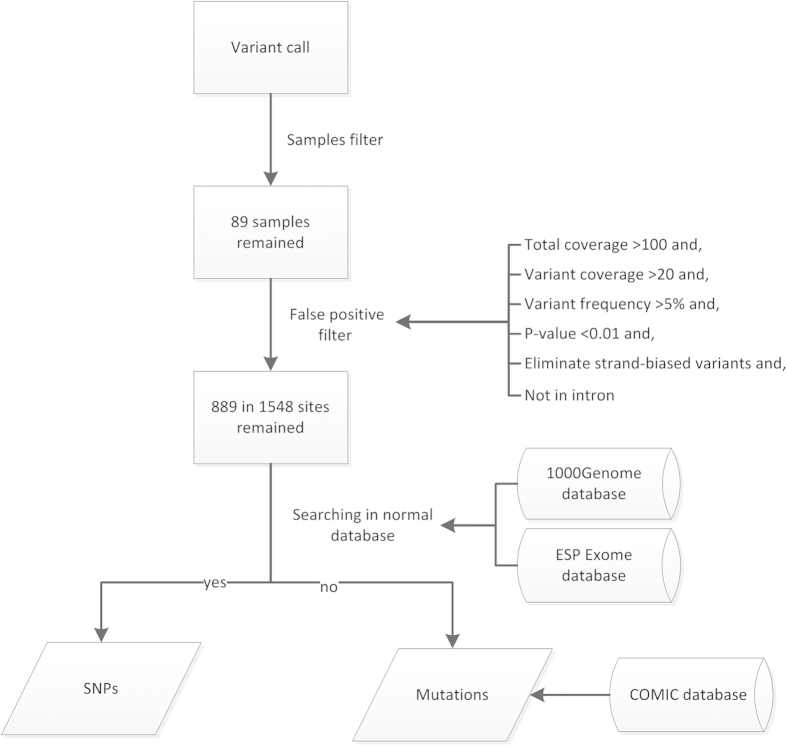
The workflow of data analysis for NGS. Data obtained from PGM runs were processed initially using Ion Torrent platform specific software. A total of 89 samples were obtained for mutation analysis.

The Ion Torrent platform was used to screen for gene mutations in 89 cases of lung adenocarcinoma metastatic lymph node specimens obtained by FNAC. Figure 1 depicts the procedure of mutation analysis assay using the Ion Torrent platform. Data obtained from the PGM runs were initially processed using Ion Torrent platform specific software, and then the “variant caller” program, which is a plug-in of Torrent Suite Software, was performed for the initial variant calling of the Ion AmpliSeq sequencing data. To obtain results more accurately, the final data were filtered through several steps. In the first step, samples with a total target coverage of 100 x < 85% were removed. In the second step, false positive sites were removed under the following conditions: a mean total coverage depth >100, each variant coverage >20, a variant frequency of each sample >5, and P-value <0.01, not in intron, and not strand-biased variants, by using the Integrative Genomics Viewer (IGV) software. Then, these variants were searched in normal database to distinguish between SNPs and mutations. Finally, mutations were searched in COSMIC database. The Sequence read distribution across 207 amplicons was normalized to 357,000 reads per sample, and the mean number of reads of each amplicon was 1062 times.

### EGFR mutation analysis in FNAC specimens with lung adenocarcinoma

**Figure 2:**
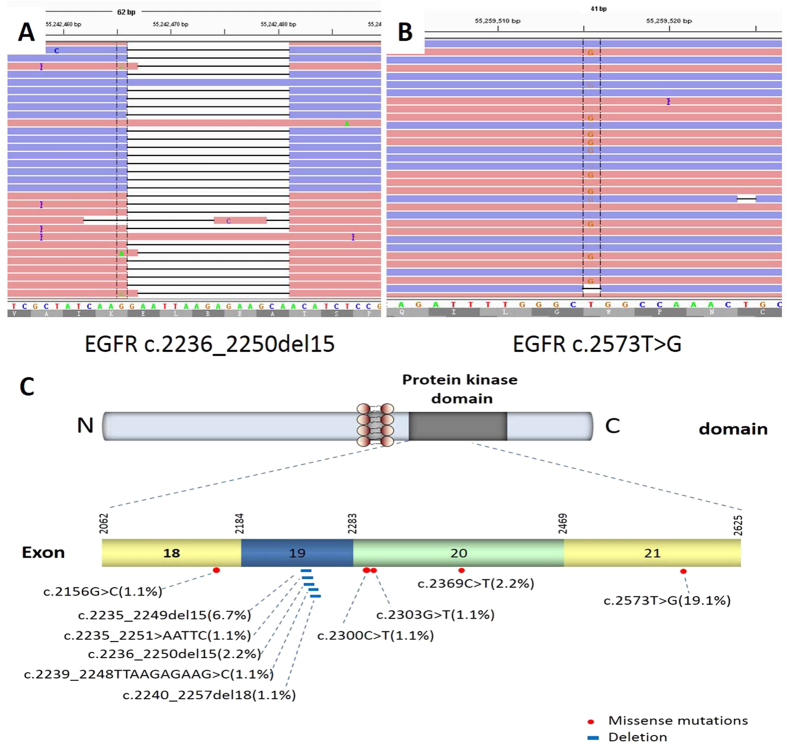
*EGFR* mutations in FNAC specimens with lung adenocarcinoma. Representative images of the reads aligned to the reference genome as provided by the Integrative Genomics Viewer (A,B). Distribution of the mutations in the kinase domain of *EGFR* (C).

Of the 89 samples, 30 (34%) were found to harbor EGFR kinase domain mutations, and 3 (3%) were found to have multiple complex mutations (Table 1). Among them, 1 was G719A (c.2156G > C) missense point mutation in exon 18, 11 were deletions in exon 19, 4 were missense point mutation in exon 20 and 17 were L858R (c. 2573T > G) missense point mutation in exon 21. Of the 11 patients with the exon 19 deletion, 8 (73%) had the commonest 15-bp deletions (delE746_A750), and 3 (27%) had other deletions. Of the 4 patients with exon 20 mutations, 2 (50%) had T790M (c.2369C > T) mutation, 1 (25%) had S768I (c.2303G > T) mutation, and 1 had A767V (c.2300C > T) mutation (Fig. 2).

### Mutation spectra in *KRAS, NRAS, BRAF, and PIK3CA*

**Figure 3:**
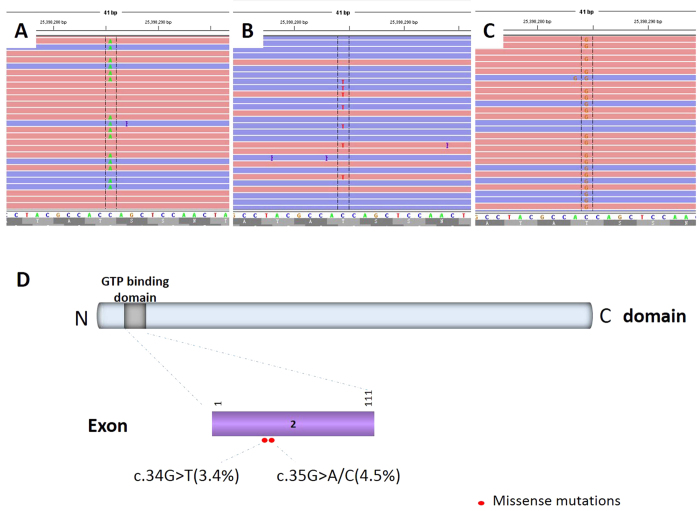
*KRAS* mutations in FNAC specimens with lung adenocarcinoma. Representative images of the reads aligned to the reference genome as provided by the Integrative Genomics Viewer (A,B,C). Distribution of the *KRAS* mutations in this study (D).

Of the 89 samples, 7 (8%) harbored *KRAS* mutations at codon 12, including 3 cases of G12C (c.34G > T) mutation, 3 cases of G12A (c.35G > C) mutation and 1 case of G12D (c.35G > A) mutation (Fig. 3). Three (3%) samples had *BRAF* mutations involving exon 11 (G469A) and exon 15 (V600E). Eight (9%) samples harbored *PIK3CA* mutations, of which 5 occurred in exon 9 and 3 in exon 20. One (1%) sample had a *HRAS* G12C mutation.

### Other mutations

Thirty-two (36%) samples harbored *TP53* mutations, including 12 in exon 5, 3 in exon 6, 8 in exon 7, 5 in exon 8 and 5 in exon 10. Of the 32 *TP53* mutation samples, 1 had multiple complex mutations. Other genes including *APC, ATM, MET, PTPN11, GNAS, HRAS, RB1, SMAD4 and STK11* were found each in one case.

## Discussion

In this study, we performed NGS to detect gene mutations in lung adenocarcinoma by using the Ion Torrent platform, demonstrating that the NGS technique had a promising potential use for multi-gene identification. The major advantage of NGS is its ability to produce an enormous volume of data less costly^14^.

The paraffin-embedded tissue has been considered optimal sample for molecular studies. Consequently, *EGFR* analysis in most published studies depended on biopsy specimens. However, biopsies are not always available, because the diagnosis of lung cancer sometimes depends on metastatic lymph node specimens obtained by FNAC through minimally invasive procedures in order to avoid more invasive techniques^17^,^18^,^19^ Therefore, the possibility of performing molecular methods on metastatic lymph node of FNAC samples is appealing^20^. Methods to detect *EGFR* or other gene mutations in specimens with an unfavorable tumor cell content need to be very sensitive^21^. The development of the NGS method represents one of the more significant technical advances in molecular biology^14^,^22^.

Some driver gene mutations are promising diagnostic and prognostic markers for lung adenocarcinomas and also represent potential therapeutic targets. Therefore, an accurate and reliable screening method for gene mutations is highly desired. To the best of our knowledge, the present study is a large series analyzing gene mutations in cytological samples. The Ion Torrent platform was performed to screen for mutations in 89 cases of lung adenocarcinoma metastatic lymph node specimens obtained by FNAC. Although the Sanger sequencing technique is the standard method to detect gene mutations, it requires a high tumor content and more starting material. NGS using the Ion Torrent technology only requires 10ng DNA for analysis. Our study has demonstrated that NGS using the Ion Torrent technology is a useful tool for gene mutation screening in lung adenocarcinoma, and may promote the development of new targeted therapies in lung adenocarcinoma patients.

## Methods

### Patients

Data in this retrospective study were analyzed anonymously. No images and private information of the patients were released. The medical ethical committee of the Cancer Hospital of the Chinese Academy of Medical Sciences (CAMS), approved the study protocol and agreed to waive the need for consent by the patients. A total of 89 Chinese patients with lung adenocarcinoma were enrolled in this study and treated at the CAMS Cancer Hospital between July 2012 and June 2013. All the lymph node specimens in these patients were obtained by fine-needle aspiration cytology (FNAC), fixed in 10% neutral buffered formalin, embedded in paraffin and finally diagnosed as metastatic lung adenocarcinoma.

### DNA preparation

DNA was extracted from paraffin-embedded tumor tissues using QIAamp® DNA Mini Kit (Qiagen, Germany), according to the manufacturer’s instructions. The quality and concentration of the DNA samples were examined by NanoDrop (Thermo).

### Next-generation sequencing (NGS)

NGS was performed using the Ion Torrent platform (Life Technologies), according to the manufacturer’s specifications. An Ion Torrent adapter-ligated library was constructed with the Ion AmpliSeq Library Kit 2.0 (Life Technologies) following the manufacturer’s protocol. Briefly, 10 ng of pooled amplicons were end-repaired, and DNA ligase was used to ligate Ion Torrent adapters P1 and A. The adapter-ligated products were purified with AMPure beads (Beckman Coulter), and the purified products were nick-translated and PCR-amplified for a total of 5 cycles. The resulting library was again purified with AMPure beads, and the size and concentration of the library were determined by Agilent 2100 Bioanalyzer and Agilent Bioanalyzer DNA High-Sensitivity LabChip (Agilent Technologies).

## Additional Information

**How to cite this article**: Qiu, T. *et al.* Next-generation sequencing for molecular diagnosis of lung adenocarcinoma specimens obtained by fine needle aspiration cytology. *Sci. Rep.* **5**, 11317; doi: 10.1038/srep11317 (2015).

## Figures and Tables

**Table 1:** Mutations found in 89 lung adenocarcinoma cytology specimens using the Ion Torrent NGS.

**Gene Mutations**	**exon**	**Number of samples with this mutation site**	**Number of samples with this mutation gene**	**Mutation Frequency**
*APC* c.4348C > T	15	1	1	1%
*ATM* c.9022C > T	62	1	1	1%
*BRAF* c.1406G > C	11	2	3	3%
*BRAF* c.1799T > A	15	1		
*CTNNB1* c.110C > T	3	2		
*CTNNB1* c.121A > G	3	1	5	6%
*CTNNB1* c.134C > T	3	2		
*EGFR* c.2156G > C	18	1		
*EGFR* c.2235_2249del15	19	6		
*EGFR* c.2235_2251GGAATTAAGAGAAGCAA > AATTC	19	1		
*EGFR* c.2236_2250del15	19	2		
*EGFR* c.2239_2248TTAAGAGAAG > C	19	1	30	34%
*EGFR* c.2240_2257del18	19	1		
*EGFR* c.2300C > T	20	1		
*EGFR* c.2303G > T	20	1		
*EGFR* c.2369C > T	20	2		
*EGFR* c.2573T > G	21	17		
*GNAS* c.601C > A	8	1	1	1%
*HRAS* c.34G > T	2	1	1	1%
*KRAS* c.34G > T	2	3		
*KRAS* c.35G > A	2	1	7	8%
*KRAS* c.35G > C	2	3		
*MET* c.3334C > T	16	1	1	1%
*PIK3CA* c.1624G > A	9	1		
*PIK3CA* c.1624G > C	9	3		
*PIK3CA* c.1633G > A	9	1	8	9%
*PIK3CA* c.3140A > G	20	2		
*PIK3CA* c.3145G > C	20	1		
*PTPN11* c.226G > A	3	1	1	1%
*RB1* c.1666C > T	17	1	1	1%
*SMAD4* c.353C > T	2	1	1	1%
*STK11* c.580G > T	4	1	1	1%
*TP53* c.1010G > T	10	2	32	36%
*TP53* c.1015G > T	10	2		
*TP53* c.1027G > T	10	1		
*TP53* c.394A > G	5	1		
*TP53* c.473G > C	5	1		
*TP53* c.473G > T	5	1		
*TP53* c.477C > T	5	2		
*TP53* c.487T > G	5	1		
*TP53* c.503A > G	5	1		
*TP53* c.524G > A	5	1		
*TP53* c.527G > A	5	1		
*TP53* c.527G > T	5	1		
*TP53* c.536A > G	5	1		
*TP53* c.541C > T	5	1		
*TP53* c.637C > T	6	1		
*TP53* c.641A > G	6	1		
*TP53* c.646G > A	6	1		
*TP53* c.725G > T	7	1		
*TP53* c.730G > T	7	1		
*TP53* c.733G > T	7	2		
*TP53* c.734G > A	7	1		
*TP53* c.734G > T	7	1		
*TP53* c.742C > T	7	1		
*TP53* c.747G > T	7	1		
*TP53* c.804C > T	8	1		
*TP53* c.818G > T	8	1		
*TP53* c.821T > C	8	1		
*TP53* c.825T > G	8	1		
*TP53* c.832C > T	8	1		

## References

[b1] Parkin D. M., Pisani P. & Ferlay J. Global cancer statistics. CA Cancer J Clin 49, 33–64 (1999). 10200776 10.3322/canjclin.49.1.33

[b2] Parkin D. M., Bray F., Ferlay J. & Pisani P. Global cancer statistics, 2002. CA Cancer J Clin 55, 74–108 (2005). 15761078 10.3322/canjclin.55.2.74

[b3] Jemal A. *et al.* Global cancer statistics. CA Cancer J Clin 61, 69–90 (2011). 21296855 10.3322/caac.20107

[b4] Schiller J. H. *et al.* Comparison of four chemotherapy regimens for advanced non-small-cell lung cancer. N Engl J Med 346, 92–98 (2002). 11784875 10.1056/NEJMoa011954

[b5] Leighl N. B. *et al.* Molecular testing for selection of patients with lung cancer for epidermal growth factor receptor and anaplastic lymphoma kinase tyrosine kinase inhibitors: American Society of Clinical Oncology endorsement of the College of American Pathologists/International Association for the study of lung cancer/association for molecular pathology guideline. J Clin Oncol 32, 3673–3679 (2014). 25311215 10.1200/JCO.2014.57.3055 PMC5321089

[b6] Kancha R. K., von Bubnoff N., Peschel C. & Duyster J. Functional analysis of epidermal growth factor receptor (EGFR) mutations and potential implications for EGFR targeted therapy. Clin Cancer Res 15, 460–467 (2009). 19147750 10.1158/1078-0432.CCR-08-1757

[b7] Shepherd F. A. *et al.* Erlotinib in previously treated non-small-cell lung cancer. N Engl J Med 353, 123–132 (2005). 16014882 10.1056/NEJMoa050753

[b8] Natale R. B. *et al.* Phase III trial of vandetanib compared with erlotinib in patients with previously treated advanced non-small-cell lung cancer. J Clin Oncol 29, 1059–1066 (2011). 21282542 10.1200/JCO.2010.28.5981

[b9] Maemondo M. *et al.* Gefitinib or chemotherapy for non-small-cell lung cancer with mutated EGFR. N Engl J Med 362, 2380–2388 (2010). 20573926 10.1056/NEJMoa0909530

[b10] Peng L., Song Z. G. & Jiao S. C. Efficacy analysis of tyrosine kinase inhibitors on rare non-small cell lung cancer patients harboring complex EGFR mutations. Sci Rep 4, 6104 (2014). 25130612 10.1038/srep06104 PMC4135336

[b11] Lynch T. J. *et al.* Activating mutations in the epidermal growth factor receptor underlying responsiveness of non-small-cell lung cancer to gefitinib. N Engl J Med 350, 2129–2139 (2004). 15118073 10.1056/NEJMoa040938

[b12] Paez J. G. *et al.* EGFR mutations in lung cancer: correlation with clinical response to gefitinib therapy. Science 304, 1497–1500 (2004). 15118125 10.1126/science.1099314

[b13] Fassina A. *et al.* Fine needle aspiration of non-small cell lung cancer: current state and future perspective. Cytopathology 23, 213–219 (2012). 22805511 10.1111/j.1365-2303.2012.01005.x

[b14] Metzker M. L. Sequencing technologies - the next generation. Nat Rev Genet 11, 31–46 (2010). 19997069 10.1038/nrg2626

[b15] Han J. Y. *et al.* Comparison of targeted next-generation sequencing with conventional sequencing for predicting the responsiveness to epidermal growth factor receptor-tyrosine kinase inhibitor (EGFR-TKI) therapy in never-smokers with lung adenocarcinoma. Lung Cancer 85, 161–167 (2014). 24857785 10.1016/j.lungcan.2014.04.009

[b16] Ross J. S. *et al.* Next-generation sequencing reveals frequent consistent genomic alterations in small cell undifferentiated lung cancer. J Clin Pathol 67, 772–776 (2014). 24978188 10.1136/jclinpath-2014-202447 PMC4145440

[b17] Lozano M. D. *et al.* Assessment of epidermal growth factor receptor and K-ras mutation status in cytological stained smears of non-small cell lung cancer patients: correlation with clinical outcomes. Oncologist 16, 877–885 (2011). 21572125 10.1634/theoncologist.2010-0155 PMC3228207

[b18] Stigt J. A. *et al.* Comparison of EUS-guided fine needle aspiration and integrated PET-CT in restaging after treatment for locally advanced non-small cell lung cancer. Lung Cancer 66, 198–204 (2009). 19231024 10.1016/j.lungcan.2009.01.013

[b19] Gu P. *et al.* Endobronchial ultrasound-guided transbronchial needle aspiration for staging of lung cancer: a systematic review and meta-analysis. Eur J Cancer 45, 1389–1396 (2009). 19124238 10.1016/j.ejca.2008.11.043

[b20] Gazdar A. F. Personalized medicine and inhibition of EGFR signaling in lung cancer. N Engl J Med 361, 1018–1020 (2009). 19692681 10.1056/NEJMe0905763 PMC3390194

[b21] Ellison G. *et al.* EGFR mutation testing in lung cancer: a review of available methods and their use for analysis of tumour tissue and cytology samples. J Clin Pathol 66, 79–89 (2013). 23172555 10.1136/jclinpath-2012-201194 PMC3582044

[b22] Kanagal-Shamanna R. *et al.* Next-generation sequencing-based multi-gene mutation profiling of solid tumors using fine needle aspiration samples: promises and challenges for routine clinical diagnostics. Mod Pathol 27, 314–327 (2014). 23907151 10.1038/modpathol.2013.122

